# Cat Eye Syndrome in a Sudanese Infant: Congenital Cataract in the Absence of Iris Coloboma: A Case Report

**DOI:** 10.1002/ccr3.72922

**Published:** 2026-06-10

**Authors:** Rayan Khalid, Imad Fadl‐Elmula

**Affiliations:** ^1^ Department of Clinical Genetics and Immunology Assafa College Khartoum Sudan; ^2^ Elite Center for Genetics Services Khartoum Sudan; ^3^ Department of Clinical Genetics, Al Neelain Stem Cell Center Al Neelain University Khartoum Sudan

**Keywords:** case report, Cat Eye Syndrome, chromosome 22q11.2, congenital cataract, small supernumerary marker chromosome, Sudan, ventricular septal defect

## Abstract

We report the first Cat Eye Syndrome case from Sudan: a 5‐month‐old female with growth retardation, craniofacial dysmorphism, congenital cataract without iris coloboma, and ventricular septal defect. Cytogenetics confirmed 47,XX,+idic(22)(q11.2). This expands CES phenotypic knowledge and underscores the need for accessible genetic diagnostics in underrepresented African populations.

## Key Clinical Message

Cat Eye Syndrome exhibits marked phenotypic heterogeneity; the hallmark iris coloboma is absent in nearly half of cases. This first Sudanese report highlights that congenital cataract and ventricular septal defect may be presenting features, emphasizing the need for early cytogenetic evaluation and multidisciplinary care in infants with multisystem dysmorphism.

## 1 Introduction

Chromosomal abnormalities represent a major group of genetic disorders contributing significantly to congenital malformations, developmental delay, and multisystem disease [1]. These alterations, whether numerical or structural, frequently manifest as recognizable syndromic conditions with variable phenotypic expression.

Cat Eye Syndrome (CES; OMIM #115470), also known as Schmid–Fraccaro syndrome, is a rare chromosomal disorder first described in 1965 and characterized by the presence of a small supernumerary marker chromosome (sSMC) derived from chromosome 22 [2]. The majority of CES cases arise de novo due to post‐zygotic nondisjunction or marker chromosome formation during early embryogenesis; however, familial transmission has been documented in rare instances, with an autosomal dominant‐like pattern of inheritance when a parent carries the supernumerary marker, conferring a recurrence risk of up to 50% [3].

The 22q11.2 critical region harbors several dosage‐sensitive developmental genes, including CECR1 (also known as ADA2), ATP6V1E1, and BCL2L13, whose overexpression is implicated in the multisystem phenotype [4]. The condition is classically associated with a triad of iris coloboma, anal anomalies, and preauricular tags or pits; however, its clinical presentation is highly heterogeneous and may involve multiple organ systems [4, 5]. Affected individuals commonly exhibit craniofacial dysmorphism, congenital heart defects, renal malformations, and skeletal abnormalities. As the classical triad is present in fewer than half of reported cases, early recognition can be challenging, particularly during infancy when phenotypic features are still evolving [4, 6].

The estimated incidence of CES ranges from 1 in 50,000 to 1 in 150,000 live births, though the true prevalence is likely underestimated due to underdiagnosis of mild or atypical presentations [7, 8]. While numerous cases have been reported in Europe, Asia, and North America, CES remains sparsely documented in Africa, reflecting both its rarity and limited access to cytogenetic diagnostic services across the continent.

Definitive diagnosis relies on cytogenetic analysis, including conventional karyotyping and molecular techniques such as chromosomal microarray analysis [4]. However, the limited availability of specialized genetic testing in many African countries may delay confirmation and appropriate genetic counseling, underscoring the need for clinical recognition to enable timely referral.

Herein, we report a case of Cat Eye Syndrome in a 5‐month‐old Sudanese infant presenting with multiple craniofacial anomalies and a congenital cardiac defect. To the best of our knowledge, this represents the first reported case from Sudan, with only a limited number of cases documented in Africa. This report aims to expand the clinical literature on CES within the African context and highlight the importance of early recognition and multidisciplinary management.

## 2 Case History/Examination

A 5‐month‐old female infant was brought to the Elite Center for Genetic Services by her parents with concerns of growth retardation and multiple dysmorphic features. She was born to non‐consanguineous parents, with the father aged 31 years and the mother 25 years at the time of delivery. The family history was unremarkable, with no history of miscarriages, stillbirths, or similar congenital conditions. The patient has two healthy siblings: a 4‐year‐old brother and a 2‐year‐old sister. She was the product of an uncomplicated pregnancy and was delivered at home via normal spontaneous vaginal delivery.

On examination, her length and weight were 58 cm and 5.2 kg, respectively. Her head circumference was 38 cm, consistent with microcephaly. All anthropometric measurements were below the 3rd centile for age according to WHO growth standards.

**FIGURE 1:**
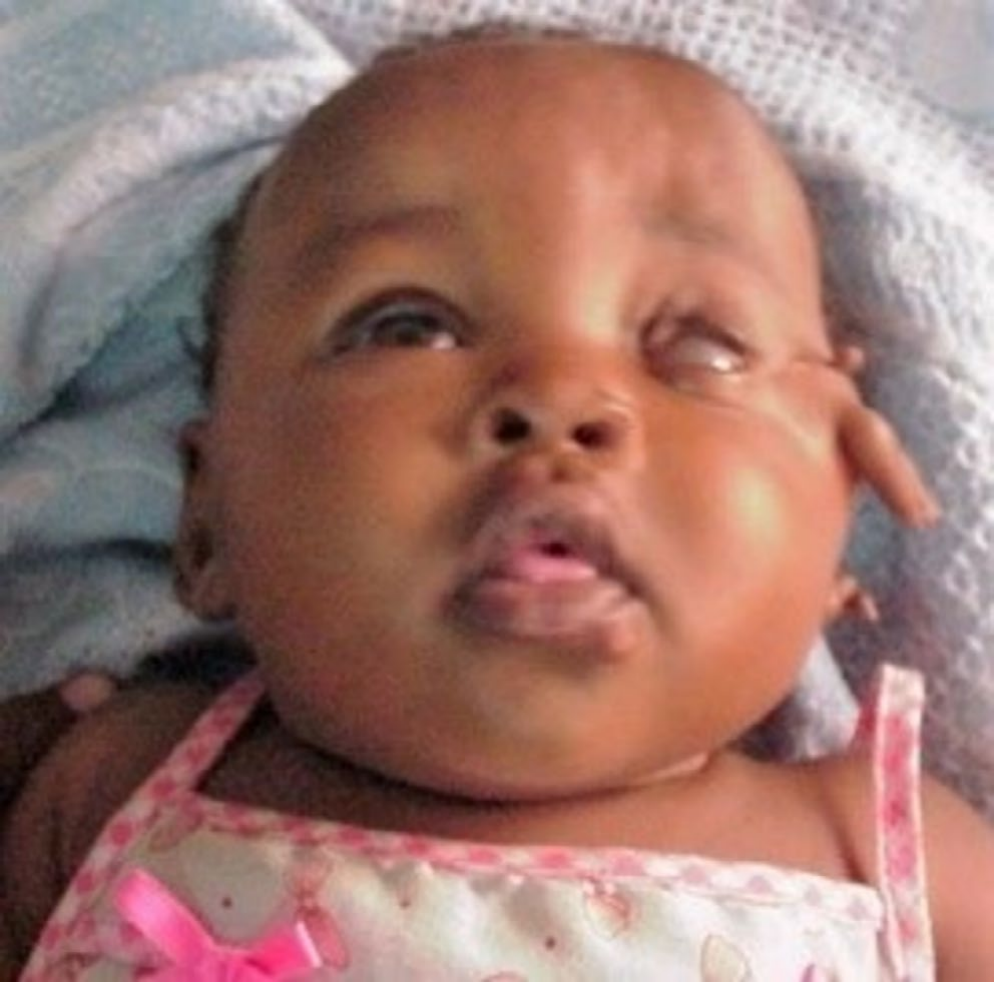
Clinical features of Cat Eye Syndrome in a 5‐month‐old female infant. Facial asymmetry with left microtia, multiple preauricular skin tags, left eye congenital cataract.

Physical examination revealed multisystem involvement. Craniofacial dysmorphic features included microcephaly, epicanthal folds, hypertelorism, a depressed nasal bridge, and a long philtrum. Ocular examination revealed a left‐sided lenticular opacity consistent with congenital cataract; notably, no iris coloboma was observed. Auricular findings comprised complete agenesis of the left external ear (microtia) with multiple preauricular skin tags and low‐set contralateral ear positioning. Neck and trunk examination demonstrated a short neck and widely spaced nipples. Limb examination revealed clenched hands (Figure 1).

Developmental assessment at 5 months of age revealed age‐appropriate gross motor milestones, including steady head control and spontaneous rolling, alongside intact visual tracking and a responsive social smile, with no evidence of developmental delay or regression.

## 3 Differential Diagnosis, Investigations, and Treatment

### 3.1 Investigations

**FIGURE 2:**
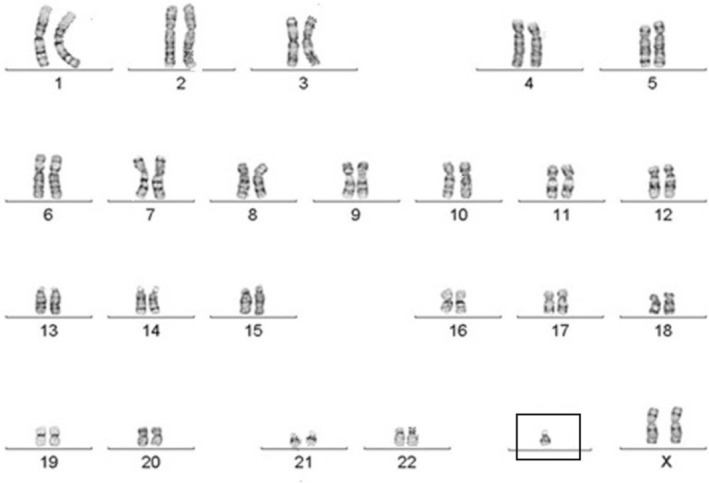
Cytogenetic analysis confirming Cat Eye Syndrome. Karyotype of peripheral blood lymphocytes showing 47,XX,+idic(22)(q11.2), consistent with a supernumerary marker chromosome derived from chromosome 22.

Routine hematological and biochemical investigations were within normal limits. Transthoracic echocardiography revealed a ventricular septal defect (VSD). Cytogenetic analysis of peripheral blood lymphocytes, performed using conventional G‐banding, in accordance with the International System for Human Cytogenomic Nomenclature (ISCN 2020) [9], demonstrated a karyotype of 47,XX,+idic(22)(q11.2) (Figure 2). FISH and chromosomal microarray analysis were not performed due to resource constraints, limiting precise breakpoint mapping and copy‐number determination. Parental karyotyping was offered to evaluate for familial transmission or mosaicism but was declined due to logistical and financial constraints.

### 3.2 Differential Diagnosis

The combination of hemifacial microsomia, unilateral microtia, and preauricular tags closely resembles oculo‐auriculo‐vertebral spectrum (OAVS). However, OAVS is typically a sporadic, non‐chromosomal condition that does not involve the supernumerary marker chromosome or the specific congenital heart defects characteristic of CES. Cytogenetic confirmation effectively distinguishes CES from OAVS in this patient.

CHARGE syndrome is another important consideration given the overlapping presentation of ear anomalies, congenital heart defects, and ocular findings. CHARGE syndrome is clinically distinguished by choanal atresia, characteristic temporal bone malformations, cranial nerve dysfunction, and pathogenic variants in the CHD7 gene. The absence of choanal atresia, the lack of typical CHD7‐related cranial nerve deficits, and the confirmed presence of a 22q11.2‐derived marker chromosome exclude CHARGE syndrome.

### 3.3 Treatment and Referrals

Following confirmation of the diagnosis, the patient was referred back to her primary pediatrician for ongoing developmental surveillance and longitudinal follow‐up. Targeted referrals were made to ophthalmology for cataract evaluation and visual rehabilitation, otorhinolaryngology for auditory assessment at 6 months to evaluate both conductive and sensorineural pathways and management of microtia, and to a pediatric neurologist for developmental surveillance, as early milestones do not exclude later cognitive impairment; longitudinal follow‐up will be maintained given that intellectual disability may emerge in a subset of CES patients. Genetic counseling was provided to the parents regarding the likely de novo nature of the chromosomal abnormality, recurrence risk, and anticipated clinical course.

## 4 Discussion

Cat Eye Syndrome is a rare chromosomal disorder caused by a small supernumerary marker chromosome derived from the 22q11.2 region of chromosome 22. The condition exhibits marked genotypic and phenotypic heterogeneity, with clinical manifestations influenced by the size, copy number, and gene content of the duplicated segment. CES may present as partial trisomy or partial tetrasomy of 22q11.2, and may occur in mosaic forms or as complex rearrangements including ring chromosomes and interstitial duplications [10]. This variability contributes to a broad clinical spectrum ranging from minimally affected individuals to those with severe multisystem involvement, thereby complicating genotype–phenotype correlations [6].

In contrast to common autosomal trisomies such as trisomy 21, 18, and 13, CES is not associated with advanced maternal age. It most frequently arises de novo as a result of abnormal chromosomal recombination or marker chromosome formation during early embryogenesis, explaining its occurrence in offspring of young mothers as in this case [4]. Although familial transmission has been reported in rare cases in which a parent carries the marker chromosome, the recurrence risk remains low in de novo cases [8]. Parental karyotyping is recommended to exclude germline mosaicism or balanced rearrangements; in this case, it was offered but declined due to resource limitations [5].

The clinical features observed in this patient are consistent with previously reported CES cases, including craniofacial dysmorphism, ocular anomalies, and congenital heart disease. However, certain findings merit attention. While iris coloboma is widely considered a hallmark of CES, it is documented in fewer than half of affected individuals; consequently, the majority of patients present without this feature, as demonstrated in our case [11]. The presence of a congenital cataract in the absence of coloboma further underscores the marked ocular variability of the syndrome. Additionally, our patient was diagnosed with a ventricular septal defect (VSD), which aligns with existing literature identifying VSD, atrial septal defects, and tetralogy of Fallot as the most frequently reported cardiac anomalies in CES [12]. The absence of cyanosis at presentation is consistent with an isolated VSD with left‐to‐right shunting in infancy.

Traditionally, CES is classified into Type I and II based on breakpoint locations within the low‐copy repeats (LCR22) and resulting copy‐number status, rather than duplication extent alone [5]. This structural distinction correlates with phenotypic severity, as Type II cases frequently exhibit more complex cardiac, renal, and neurological involvement. Although CES is fundamentally a gene dosage‐increase disorder, the 22q11.2 region is genomically complex and harbors several dosage‐sensitive developmental genes. Consequently, phenotypic overlap with 22q11.2 microdeletion syndromes (such as DiGeorge or velocardiofacial syndrome) has been reported, likely reflecting shared pathogenic pathways disrupted by either haploinsufficiency or gene overexpression within the same critical interval [4]. The karyotype observed in this patient, 47,XX,+idic(22)(q11.2), is consistent with classic CES architecture, and the observed clinical spectrum aligns with established type‐specific genotype–phenotype correlations.

This phenotypic overlap necessitates careful consideration of differential diagnoses, particularly OAVS and CHARGE syndrome. The patient's presentation of unilateral microtia, multiple preauricular tags, and hemifacial microsomia strongly overlaps with OAVS, and clinically, this child would more readily be assigned to the OAVS spectrum. However, OAVS is typically non‐chromosomal and lacks iris coloboma or characteristic 22q11.2 rearrangements [13]. CHARGE syndrome, associated with pathogenic variants in the *CHD7* gene, includes coloboma, choanal atresia, ear anomalies, and congenital heart defects, but also features characteristic temporal bone anomalies and vestibulocochlear dysfunction [14]. In this case, the identification of the idic(22)(q11.2) marker definitively establishes CES, highlighting the critical role of cytogenetic analysis in differentiating clinically overlapping conditions, particularly in settings where molecular testing is unavailable.

Despite recognizable features, diagnosing CES remains challenging due to phenotypic variability and limited availability of specialized genetic services, particularly in African healthcare settings. While conventional karyotyping established the diagnosis in our patient, it may not detect small or structurally complex marker chromosomes. Advanced molecular techniques provide higher‐resolution characterization of these abnormalities and refine genotype–phenotype correlations [15]. However, the high cost and limited accessibility of these technologies in Sudan and similar regions pose significant barriers to early diagnosis and comprehensive genetic characterization, potentially complicating timely multidisciplinary management.

Although more than 300 cases of CES have been reported worldwide, documentation from African populations remains extremely limited. A systematic literature search across PubMed, Scopus, and Orphanet (search terms: “Cat Eye Syndrome”, “22q11.2 duplication”, “supernumerary marker chromosome 22”, “Africa”, “Sudan”; coverage: 1965–2026) yielded no prior documented cases from Sudan. This disparity likely reflects both underdiagnosis and underreporting. To the best of our knowledge, this represents the first reported case of CES from Sudan, contributing valuable clinical and cytogenetic data from an underrepresented population. The co‐occurrence of congenital cataract without iris coloboma, alongside a VSD and characteristic craniofacial dysmorphism, further expands the recognized phenotypic spectrum of the syndrome.

Management of CES is inherently complex due to its multisystem involvement and requires a coordinated multidisciplinary approach. In this patient, the VSD necessitates routine cardiological monitoring for potential spontaneous closure or future surgical intervention, while the congenital cataract requires prompt ophthalmological evaluation to prevent amblyopia and optimize visual development. Auricular anomalies warrant audiological assessment and long‐term reconstructive planning. In many African settings, however, fragmented healthcare systems, limited access to subspecialty care, and financial constraints complicate long‐term follow‐up and continuity of care [16]. Strengthening pediatric genetic services, improving referral pathways, and promoting multidisciplinary collaboration are essential steps toward optimizing outcomes for affected individuals. Genetic counseling regarding recurrence risk, developmental expectations, and coordinated care planning remains essential, yet consistently delivering these services in resource‐limited settings remains a significant logistical challenge.

## Author Contributions

**Rayan Khalid:** conceptualization, investigation, methodology, data curation, writing – original draft, project administration, formal analysis, validation, software. **Imad Fadl‐Elmula:** conceptualization, validation, visualization, supervision, project administration, writing – review and editing.

## Funding

The authors have nothing to report.

## Ethics Statement

Ethical approval for this case report was obtained from the Institutional Review Board of Assafa College (Approval ID: DSR‐AC EC NO 22–8‐11). Written informed consent was obtained from the patient's parents for publication of clinical details, photographs, and genetic findings.

## Consent

Written informed consent was obtained from the patient's parents for publication of this case report and any accompanying images.

## Conflicts of Interest

The authors declare no conflicts of interest.

## Data Availability

Data from this case are available upon request.
